# President’s Address, Central Texas District Medical Society, Section on Obstetrics and Gynecology*Read at the recent meeting of Central Texas District Medical Society at Temple, Texas.

**Published:** 1907-11

**Authors:** W. L. Crosthwaite

**Affiliations:** Holland, Texas


For the Texas Medical Journal.

### President's Address, Central Texas District Medical Society, Section on Obstetrics and Gynecology.*

W. L. CROSTHWAITE, M. D., HOLLAND, TEXAS.

Let me preface my address with an apology for whatever of incongruity of thought or force of diction in this brief address is discoverable by the light of more forcible reasoning than I am at this time capable of, having so recently undergone an operation for acute suppurative mastoiditis.

The field of medical research is constantly broadening and each day some valuable and practicable truth is revealed, thus constantly enriching our store of scientific knowledge. To casually mention all that has been done in the departments of obstetrics and gynecology during the past year would seem tedious and uninteresting. I shall not attempt a review of the great advancements we have made, but rather add one thought, and that in the form of a criticism. In all our material progress in the science of obstetrics and gynecology we have devoted seemingly more attention to effects than we have to causes.

The art of operative surgery has rapidly sped to perfection, our knowledge of bacteria and opsonins is almost perfect, the science of disease has been made plain through the tireless efforts of our

---

*Read at the recent meeting of Central Texas District Medical Society at Temple, Texas.

pathologists. Likewise the science of applied therapeutics has advanced far beyond the stage of empiricisms and experimental medicine. In short, we have made such progress in every department of medicine and surgery that we may with becoming grace boast of it, but shall we be satisfied until every possibility of preventative medicine has been thoroughly investigated and until prophylaxis becomes a practical and universally applied science.

Prophylaxis in obstetrical practice should begin early. I mean that the family physician should be the guide of the child from infancy through the various stages of life to womanhood. The health of the young girl should be carefully guarded with the view of preserving the integrity of her sexual and reproductive functions. Many of the complications of pregnancy and many of causes of gynecological conditions have their origin in the indiscretions in mode of life, manner of dress and environments of early life. The factitiousness of our modern society has been productive of a race of women with marked constitutional predispositions to many if not all of the pathological conditions usually classed as "diseases peculiar to their sex," thus affording a constantly broadening field for the gynecologist and, as well, an increasing load of trouble for the obstetrician.

There can be no question as to who is to blame for such conditions. The mothers can not in all cases properly instruct their daughters because they themselves have not been properly instructed. They can not properly guard their daughters through the trying period of adolescence because of their lack of knowledge of the vital laws of hygiene and of their misconception of the psychological manifestations and physiological phenomenon of the girl, maiden and woman. The work of redeeming womankind from that state of hereditary invalidism to which they are speedily drifting must belong and be accomplished not by the physician in the capacity of surgeon, gynecologist or obstetrician, but by the physician in the rôle of the "good old family doctor," who does not think it beneath his dignity to talk heart to heart with mothers, imparting through them to their daughters such knowledge as is essential to normal development and perfect health.

Passing from the speculative to the practical part of our subject, I want to urge that it is the duty of obstetricians to educate the laity up to that point where it will be thought absolutely imperative for every pregnant woman to be placed under the care of her physician as soon as conception is known to have taken place. This is the inherent right of every woman and the duty of every family physician to insist that she have that right. For a careful attention to prophylaxis on the part of the obstetrician is of the greatest value in anticipating and warding off the dangerous complications of the pregnant, parturient and puerperal state, and in preventing the many subsequent disabilities of a gynecological nature.

I do not mean that we should regard or lead our patients to consider the pregnant state as a condition of chronic invalidism. This mistake, perhaps, is too often made, thus leading to neglected exercise and proper nourishment. Normal pregnancies are physiological processes, but the obstetrician who has carefully studied his cases will insist, and rightly, too, that many pregnancies are pathological in their nature.

The woman during the period of physiological pregnancy should have proper advice as to diet, exercise, fresh air, clothing, care of skin and breasts and time and amount of rest and sleep, her physician should carefully look after the condition of her mental condition, alimentary and urinary tract. The woman during a period of pathological pregnancy must have the constant attention of her physician. This is obvious when we consider the varied pathology which it may not be amiss to mention, beginning with (1) diseases of the deciduæ, and following the classification of Edgar we find no less than twenty-seven classes of pathological pregnancy with from two to twenty-four subdivisions under each class. Diseases of the chorion; anomalies of the amnion, the umbilicus and the chord; deformities, antenatal disease and death of the fetus; diseases of the genital organs, of the alimentary tract, of the urinary tract and of the nervous, respiratory, circulatory and osseous systems; infectious and skin disease; ectopic gestation; abortion; fever of pregnancy, and the toxemias of pregnancy, are the most common.

It is not the intention of this paper to deal with any of these conditions in detail, but rather to emphasize the first thought and that it is to direct our efforts more in the future toward detecting and eliminating causes than towards attempting to heal or alleviate effects.

Let our greatest and most zealous work be along the lines of preventative medicine.

---

If a patient presents himself with a painless cellulitis of the finger or hand, it is necessary to make a careful examination for the possible presence of syringomyelia.—*American Journal of Surgery.*

